# Omics-based Approach Towards Macrophages: New Perspectives of Biology and Function in the Normal and Diseased Heart

**DOI:** 10.7150/ijbs.112061

**Published:** 2025-05-27

**Authors:** Chao Yang, Huajun Li, Yuxing Chen, Wei Zhu, Jian'an Wang

**Affiliations:** 1 Department of Cardiology, The Second Affiliated Hospital, College of Medicine, Zhejiang University, Hangzhou 310009, China; 2 State Key Laboratory of Transvascular Implantation Devices, Hangzhou 310009, China; 3 Heart Regeneration and Repair Key Laboratory of Zhejiang province, Hangzhou 310009, China; 4 Transvascular Implantation Devices Research Institute (TIDRI), Hangzhou 310053, China

**Keywords:** macrophage, cardiovascular disease, multi-omics, single-cell, spatial transcriptomics, metabolomics

## Abstract

Macrophages play a crucial role not only in maintaining homeostasis but also in initiating inflammatory responses to various forms of stress or injury, thereby contributing to tissue damage while concurrently promoting recovery. Furthermore, the diversity of macrophage subtypes, their spatial distribution, and distinct cellular functions are closely linked to the pathogenesis and severity of cardiovascular diseases such as myocardial infarction, atherosclerosis, heart failure, and myocarditis. This association underscores the importance of investigating macrophage heterogeneity in different pathological contexts. Recent advances in multi-omics technologies—including single-cell RNA sequencing, spatial transcriptomics, and metabolomics—have elucidated the heterogeneity of macrophages, their intercellular interactions, underlying functional mechanisms, and spatial organization. In this review, we systematically summarize the diverse phenotypes and functional plasticity of macrophages in the regulation of cardiovascular diseases, with particular emphasis on the novel insights afforded by multi-omics approaches. We focus on the characteristics of macrophages in both physiological and pathological states, thereby providing reference points for clinical macrophage-targeted strategies and their translational significance.

## Introduction

Macrophages have long been recognized for their complex interactions within the cardiovascular system, serving as the most abundant immune cells in cardiac tissue. They play pivotal roles in both maintaining homeostasis and contributing to the development of cardiovascular diseases (CVD) 1-3. As key regulators of post-injury inflammation and the local microenvironment, their residency and polarization are closely associated with disease progression 4. Macrophages are essential in orchestrating phagocytosis, immune surveillance, inflammation, and cardiovascular remodeling. Following injury, they are actively recruited to damaged areas, where they become the predominant immune cells, clearing tissue debris through phagocytosis and releasing substantial amounts of pro-inflammatory cytokines and proteases 5,6. Furthermore, macrophages secrete a diverse array of mediators that promote extracellular matrix (ECM) deposition, cell proliferation, and angiogenesis, while also modulating immune responses and fibrosis via interactions with other cell types 7.

Although macrophages share common features, their functional phenotypes vary according to specific disease contexts. Distinct macrophage subpopulations are distributed across various cardiovascular compartments—including myocardial and vascular tissues—demonstrating remarkable adaptability to different microenvironments. Therefore, effective treatment of CVD requires the precise identification and selective targeting of macrophage phenotypes that mediate distinct pathological processes. Recent advancements in investigative technologies—particularly multi-omics approaches such as single-cell RNA sequencing (scRNA-seq), spatial transcriptomics (ST-seq), proteomics, and metabolomics—have revealed numerous macrophage subsets 8-12. These methodologies provide unprecedented insights into the spatiotemporal heterogeneity and specialized functions of cardiovascular macrophages. Multi-omics analyses have further characterized the unique attributes of each macrophage subset under both physiological and pathological conditions 13,14. By elucidating the intricate diversity among macrophages, researchers can achieve a deeper understanding of their functional roles, advancing from broad concepts of immune processes to precise characterization of individual cellular components.

This review offers, from a multi-omics perspective, a comprehensive analysis of recent advancements in cell clustering, spatial localization, and functional heterogeneity of macrophages under homeostatic and pathological conditions—including ischemic heart injury (most commonly myocardial infarction), non-ischemic cardiac insults leading to heart failure such as myocarditis, and vascular diseases including atherosclerosis and diabetic vascular complications. By illuminating the complex biological processes mediated by macrophages, this review aims to discuss emerging therapeutic targets and novel strategies for macrophage-focused interventions in cardiovascular disease.

Please note that human genes referenced in this review follow the HUGO Gene Nomenclature Committee guidelines, with gene names represented as capitalized abbreviations. Mouse gene nomenclature adheres to the Mouse Genome Informatics conventions.

## Steady-State Macrophages

### Macrophage Metabolism and Physiological Functions

Macrophage metabolism plays a pivotal role in determining their physiological functions, as demonstrated by extensive research in immunometabolism 15. Under steady-state conditions, glucose, lipids, and glutamine constitute the principal metabolic substrates for macrophages. In response to diverse stimuli, macrophages exhibit metabolic flexibility, shifting substrate utilization and activating specific metabolic pathways 16. The accumulation of metabolic end-products and intermediates regulates macrophage phenotypes, thereby facilitating tailored responses to dynamic microenvironmental signals 17. In addition to the cell-intrinsic effects of metabolism, intercellular influences are also significant 18. Studies have shown that macrophages modulate their microenvironment and regulate organ function through the uptake and secretion of various metabolites 19-21. Therefore, investigating macrophage metabolic kinetics yields valuable insights into the regulation of their phenotypes and functional roles.

### M1/M2 Macrophage Polarization

Over recent decades, the *in vitro* M1/M2 macrophage polarization model has been widely used to investigate the interplay between immune functions and metabolism 22. Bone marrow-derived macrophages (BMDMs) are considered to be in the M0 state after treatment with colony-stimulating factors 23. Upon stimulation of Toll-like receptors (TLRs) by agonists such as lipopolysaccharide (LPS) and/or cytokines like interferon-γ (IFN-γ), macrophages polarize into the classically activated, pro-inflammatory M1 phenotype. These M1 macrophages are characterized by high expression levels of markers such as CD80, CD86, and inducible nitric oxide synthase (iNOS) 24. By contrast, alternatively activated, anti-inflammatory M2 macrophages differentiate in response to interleukin-4 (IL-4) or interleukin-13 (IL-13). M2 macrophages express high levels of markers such as CD163, CD206, ARG1, FIZZ1, and YM1, reflecting their reparative and immunoregulatory functions. M2 macrophages can be further subdivided into four subpopulations: M2a, M2b, M2c, and M2d 25. Among these, the M2b subset uniquely secretes both pro-inflammatory and anti-inflammatory factors to regulate immune responses, whereas the other subsets predominately exhibit reparative phenotypes by producing anti-inflammatory and profibrotic factors 26.

The M1 and M2 phenotypes not only perform opposing immune functions but also depend on distinct metabolic pathways. Pro-inflammatory M1 macrophages exhibit enhanced glycolysis and activation of the pentose phosphate pathway, which supports biosynthetic demands and enhances antibacterial activity 27. Conversely, M2 macrophages primarily rely on fatty acid oxidation and glutamine metabolism, along with increased mitochondrial respiration, supporting their role in inflammation resolution 28. Upon IL-4 stimulation, M2 macrophages upregulate genes associated with fatty acid metabolism 29 and polyamine synthesis from arginine 30, further contributing to their reparative functions. Although the M1/M2 polarization model offers certain advantages, it does not sufficiently reflect the complexity and functional diversity of macrophages *in vivo*, due to the influence of numerous factors, especially the broad range of changes in the microenvironment. Recent advances have redefined macrophages according to their developmental origins, classifying them as either tissue-resident or monocyte-derived, each exhibiting distinct phenotypes and functions 22,31. Differences in macrophage origin result in significant variations at the epigenetic and transcriptomic levels, which are shaped by the availability of metabolic substrates within specific organs and conditions 32. Thus, a more nuanced characterization of macrophage metabolic profiles can advance our understanding of their diverse functional roles.

Cardiovascular macrophages, like other tissue-resident macrophages, are relatively few in number yet are critical for maintaining tissue homeostasis 33-35. Importantly, metabolic changes during development or under pathological conditions influence macrophage phenotypes, which in turn modulate the tissue microenvironment and function. The advent of high-throughput technologies has enabled omics-based investigations into the metabolic characteristics of cardiovascular macrophages. Whereas earlier studies focused on whole-tissue metabolic activity, recent investigations have identified macrophage-specific metabolic pathways that are closely linked to systemic alterations 36-38.

### Single-Cell Sequencing of Cardiac Macrophages

The development of single-cell RNA sequencing (scRNA-seq) has transformed our understanding of macrophage biology. Previously, macrophages were simplistically classified as either pro-inflammatory or anti-inflammatory agents within the immune system 2; they are now recognized as a highly heterogeneous population exhibiting diverse phenotypes and functions 32,39,40. scRNA-seq has become an indispensable tool for analyzing macrophage heterogeneity at single-cell resolution. This technology has surpassed the traditional binary M1/M2 classification, revealing a complex spectrum of activation states, and enabling detailed analyses of macrophage dynamics as they adapt to intricate microenvironments 41,42.

Single-cell sequencing approaches have allowed researchers to identify distinct subsets of cardiac macrophages involved in homeostasis 43,44, without solely relying on highly specific markers or lineage tracing 45,46. Dick et al. conducted pioneering single-cell analyses of cardiac macrophages, classifying them based on CD45 and high CD64 expression into three major resident cardiac macrophage clusters 47: (1) cells with high levels of Timd4, Lyve1, and Folr2 (collectively termed TLF+), (2) macrophages with high Ccr2 expression (Ccr2^hi^), and (3) those with elevated MHCII expression (MHCII^hi^). These clusters exhibit functional parallels across diverse tissues. The TLF+ cluster is enriched for cellular transport and endocytosis pathways; the Ccr2^hi^ cluster is associated with cellular activation, degranulation, and immune responses; and the MHCII^hi^ cluster is involved in antigen presentation and other immune processes. These findings corroborate previous work by Chakarov et al. 46, who classified tissue-resident macrophages mainly into Lyve1^hi^ and MHCII^hi^ subsets. Notably, Lyve1^hi^ macrophages share a gene expression profile with the TLF+ macrophages identified by Dick et al.

Further characterization of human cardiac tissue has refined immune cell profiles across six anatomical regions of the adult heart 48, identifying resident cardiac macrophages with distinct inflammatory and protective transcriptional signatures. These include the LYVE1+FOLR2+ cluster, analogous to the murine TLF+ macrophages, and antigen-presenting macrophages expressing HLA-related genes such as HLA-DRA, HLA-DMA, HLA-DMB, and HLA-DPA1, resembling the MHCII^hi^ cluster in mice. Additionally, a unique DOCK4+ macrophage cluster expressing IL4R, STAT3, and ITGAM—but lacking C1QA or FOLR2—has also been described.

### Spatial Transcriptomics of Cardiac Macrophages

The application of spatial transcriptomics has addressed the lack of structural context inherent to single-cell technologies, enabling insights into tissue niches and specialized cell populations with distinct functions 49,50. Analysis of eight anatomical regions in the heart using spatial transcriptomics identified tissue-resident macrophages (LYVE1+) not only in the sinoatrial node (SAN), but also in the atrioventricular node (AVN) 50. Notably, the human SAN exhibits a compartmentalized structure, consisting of a central region where functionally essential P cells are intermingled with activated fibroblasts and glial cells, surrounded by a peripheral region containing immune cells—including LYVE1+ macrophages—and fibroblast populations, a configuration not observed in the AVN. In the epicardium across all four cardiac chambers, LYVE1+IGF1+ macrophages, as well as plasma B cells, are present alongside other cell types such as mesothelial cells, fibroblasts, lymphatic endothelial cells, and adipocytes. Niche analysis of immune cells in the epicardium revealed that LYVE1+IGF1+ macrophages have a pivotal role in recruiting and supporting plasma B cells, which secrete immunoglobulins and function as an immune barrier. Dysregulation of these macrophages may contribute to autoimmune mechanisms underlying heart disease 51. Another study indicated that Lyve1^lo^MHCII^hi^ macrophages predominantly localize adjacent to nerves, while Lyve1^hi^MHCII^lo^ macrophages are preferentially found near blood vessels 52.

### Origin and Development of Cardiac Macrophage Subsets

The origins of steady-state macrophage subsets have long been of interest in immunological research. Early studies indicate that resident macrophages—especially the TLF+ subset and the MHCII^hi^ / HLA-DR^hi^ CCR2- population—are initially derived primarily from the yolk sac, followed by the fetal liver during embryogenesis 53,54. After birth, adult tissue macrophages begin to receive input from, or are progressively replenished by, circulating monocytes, which primarily differentiate into CCR2+ macrophages 13,55. Transitional stages in macrophage development can be tracked using markers such as Ccr2, Csf1r, and Cx3cr1^56^. Circulating monocytes express Ccr2, a chemokine receptor important for migration, and are enriched for components of the NOD-like receptor protein 3 (NLRP3) inflammasome. Ccr2 expression reflects dynamic changes in macrophage phenotype and origin, making it a key marker for classifying cardiac macrophages. Both resident and recruited cardiac macrophages exhibit variable MHC-II/HLA-DR expression, which is crucial for antigen presentation and T-cell activation 46. Notably, MHC-II expression in embryonically derived macrophages is gradually upregulated after birth, initially appearing in the Ccr2+ subset and subsequently in the Ccr2- subset 47,57. As a result, most cardiac macrophages in neonatal mice are CCR2- MHC-II^lo^, whereas the adult mouse heart contains three distinct resident macrophage subsets 58.

## Macrophage Subsets in Various Heart Diseases

### Myocardial Infarction

Myocardial infarction (MI) causes ischemic injury, which triggers a robust inflammatory response, the influx of immune cells, and cardiac remodeling 59. Acute MI leads to the diversification, mobilization, and recruitment of both innate and adaptive immune cells to the infarcted region 60. Among these, macrophages play a central role in clearing necrotic tissue, facilitating wound healing 61,62, and orchestrating remodeling processes 63. The functions of macrophages depend on the type and proportion of subsets present at different stages—such as the acute inflammatory and chronic repair phases. Moreover, the distribution and roles of macrophage subsets differ between the infarct zone and peri-infarct region, underscoring their spatial and temporal heterogeneity during cardiac injury and repair.

#### Metabolic changes of macrophages in MI

In the acute phase, MI can be divided into two stages: the early inflammatory phase and the late resolution phase 64. Initially, hypoxia in the infarcted area leads to a metabolic switch toward glycolysis and increased lactate production. This glycolytic reprogramming is associated with the predominance and expansion of CCR2+ monocyte-derived macrophage subsets. Traditionally, glycolysis provides rapid ATP production and contributes to the generation of ribose sugars and NADPH via the pentose phosphate pathway 18, which, in turn, promotes the secretion of pro-inflammatory cytokines. This has been corroborated by the upregulation of glycolytic and hypoxia-response genes in cardiac macrophages within 24 hours post-MI 65. During ischemia, enhanced glycolysis is accompanied by suppressed oxidative phosphorylation. Concurrent inhibition of the tricarboxylic acid (TCA) cycle leads to the accumulation of succinate 66 and citrate 67. Succinate stabilizes HIF1α, further activating glycolysis 68, and upon oxidation by succinate dehydrogenase during reperfusion, promotes IL-1β production in macrophages 69. Citrate can be exported to the cytoplasm and converted to acetyl-CoA for histone acetylation 70, thereby regulating inflammatory gene expression 71.

Accompanying MI is widespread apoptosis of both myocardial and non-myocardial cells, phagocytosis of apoptotic cells triggers metabolic switching to oxidative phosphorylation, mediated by engulfed metabolites especially lipids 72. Lysosomal hydrolysis of cholesterol esters within endocytic compartments leads to the formation of anti-inflammatory oxysterols 73. Additionally, increased fatty acids promote anti-inflammatory macrophage responses by enhancing IL-10 synthesis 19 and activating PPARγ coactivator-1β (PGC-1β) 29. These metabolic shifts underlie macrophage phenotypic plasticity, resulting in the emergence of non-steady-state subsets as MI progresses 74,75. As inflammation subsides, the infarcted area is increasingly infiltrated by fibroblasts and extracellular matrix components. Restoration of blood flow via angiogenesis steers cardiac metabolism toward fatty acid oxidation, favoring the expansion and activity of anti-inflammatory or reparative macrophage subsets such as Trem2^hi^ and Bhlhe41+ macrophages 76,77. In this environment, macrophages downregulate glycolytic genes and increasingly utilize fatty acids to generate pro-resolving mediators, thereby supporting anti-inflammatory functions and tissue repair 78.

#### Spatiotemporal Dynamics of Cardiac Macrophages in MI

Following MI, there is a rapid influx of CCR2+ Ly6C^hi^ monocytes and CCR2+ monocyte-derived macrophages, which displace resident macrophages (including TLF+ and MHC-II^hi^ subsets) from ischemic regions within 48 hours 31,33. Between day 4 and day 28 post-MI, the absolute number of resident macrophages gradually increases, while the proportion of recruited macrophages declines. However, even at four weeks post-MI, the ratio of resident to recruited macrophages does not return to baseline. Jung et al. showed that, in MI mice, the proportion of resident macrophages sharply declines but begins to recover by day 3 for the MHC-II^hi^ cluster and day 5 for the TLF+ cluster, stabilizing around day 7 post-MI 79. Spatial transcriptomics sequencing (ST-seq) revealed that, on the first day after MI, macrophages are distributed throughout the heart rather than localized to the infarct region. By day 3, macrophages begin accumulating in the infarct zone, with peak abundance during the late phase 79. These data highlight the spatial and temporal heterogeneity and redistribution of macrophages throughout MI progression.

#### Functional Macrophage Subsets and Fibrotic Crosstalk

Dynamic changes in macrophage populations during the late acute phase of MI are closely linked to post-infarction repair processes 80. Distinct macrophage clusters emerge during this period 79, including interferon-responsive (IFN) clusters and proliferating clusters. Rizzo et al. identified that the infarcted heart features two pro-inflammatory macrophage clusters—Isg15^hi^ (also termed IFNIC) and MHCII+Il1b+—as well as a non-inflammatory Trem2^hi^ cluster 81. The infiltration of MHCII+Il1b+ and Isg15^hi^ macrophages peaks between days 3 and 7 post-MI, whereas Trem2^hi^ macrophages peak between days 3 and 5. Trem2^hi^ macrophages are localized to the ischemic area but are absent from remote, viable myocardium, reflecting their likely role in phagocytosis.

Amrute et al. demonstrated distinct spatial relationships between macrophage subsets and fibroblast populations in infarcted human hearts 82. CCR2+ macrophages preferentially co-localize with fibrotic FAP+/POSTN+ fibroblasts, thereby establishing an immune-fibroblast niche within the infarct core, while resident macrophages are associated with APOE+/AGT+ fibroblasts. Notably, regions enriched in FAP+/POSTN+ fibroblasts show greater infiltration of CCR2+ macrophages compared to remote areas, suggesting that local microenvironmental cues drive recruitment. Mechanistically, CCR2+ macrophages promote fibroblast activation through two principal pathways: the TGF-β/Smad3 pathway, which facilitates fibroblast migration, transdifferentiation, and extracellular matrix synthesis; and macrophage-derived IL-6, which triggers autocrine STAT3 activation in fibroblasts, further enhancing TGF-β/Smad3 signaling and promoting fibrotic remodeling. Additionally, the IL-1β/NF-κB axis drives fibroblast proliferation 83. Targeting this axis with IL-1β-neutralizing antibodies significantly reduces the abundance of FAP+ fibroblasts and improves cardiac function in experimental models, underscoring its translational therapeutic potential 82. Collectively, multi-omics findings elucidated the distribution, function, and intercellular communication of distinct macrophage subgroups within the infarcted, border, and remote zones of the heart.

#### Macrophage subsets as potential therapeutic targets for MI

Advances in single-cell sequencing have revealed distinct macrophage subsets that represent promising therapeutic targets for MI repair. In early ischemic cardiac tissue, SPP1+ macrophage clusters increase significantly. These macrophages promote inflammation and fibrosis by remodeling the extracellular matrix and activating fibroblasts through SPP1/CD44 and SPP1-αvβ3 integrin signaling pathways 82,83. Their essential role in cardiac remodeling has been validated in zebrafish studies 86. Within these SPP1+ macrophage clusters, CD36—a key receptor for phagocytosis—is upregulated and is crucial for binding and clearing apoptotic and necrotic neutrophils, thus playing a unique role in cardiac remodeling post-MI 87. Trem2^hi^ macrophages are primarily active during the later stages of MI 81. By engaging the TREM2/SYK signaling axis, these macrophages promote tissue repair and immunomodulation 88. They secrete anti-inflammatory cytokines such as IL-10 and TGF-β, reduce neutrophil infiltration, and maintain cardiomyocyte homeostasis by clearing dysfunctional mitochondria. Loss of the Trem2 gene exacerbates post-MI remodeling, while administration of soluble Trem2 improves cardiac recovery through enhanced anti-inflammatory activity 33. In human myocardial infarction samples, about half of TREM2-expressing macrophages also co-express SPP1, indicating a shared phenotype involved in tissue repair and remodeling. Rizzo et al. further identified two populations among Trem2hi macrophages: the Trem2^hi^Spp1^hi^ subset, representing an intermediate state between monocytes and macrophages, and the Trem2^hi^Gdf15^hi^ subset, corresponding to differentiated macrophages. These populations peak sequentially in the infarcted heart. Bhlhe41+ resident macrophages appear transiently in the "developing" infarct zone, which is characterized by abundant monocytes, macrophages, and myofibroblasts, while monocyte-derived Trem2^hi^ Spp1^hi^ macrophages predominate in the "old" infarct zone, where neutrophils, endothelial cells, and fibroblasts are enriched 77. Bhlhe41+ resident macrophages suppress myofibroblast activation and myocardial fibrosis, thus limiting infarct expansion following MI 76. Collectively, these findings highlight the phenotypic plasticity of macrophage subsets as spatiotemporal regulators of myocardial repair and support the development of stratified, phase-specific therapeutic strategies.

**Figure 1:**
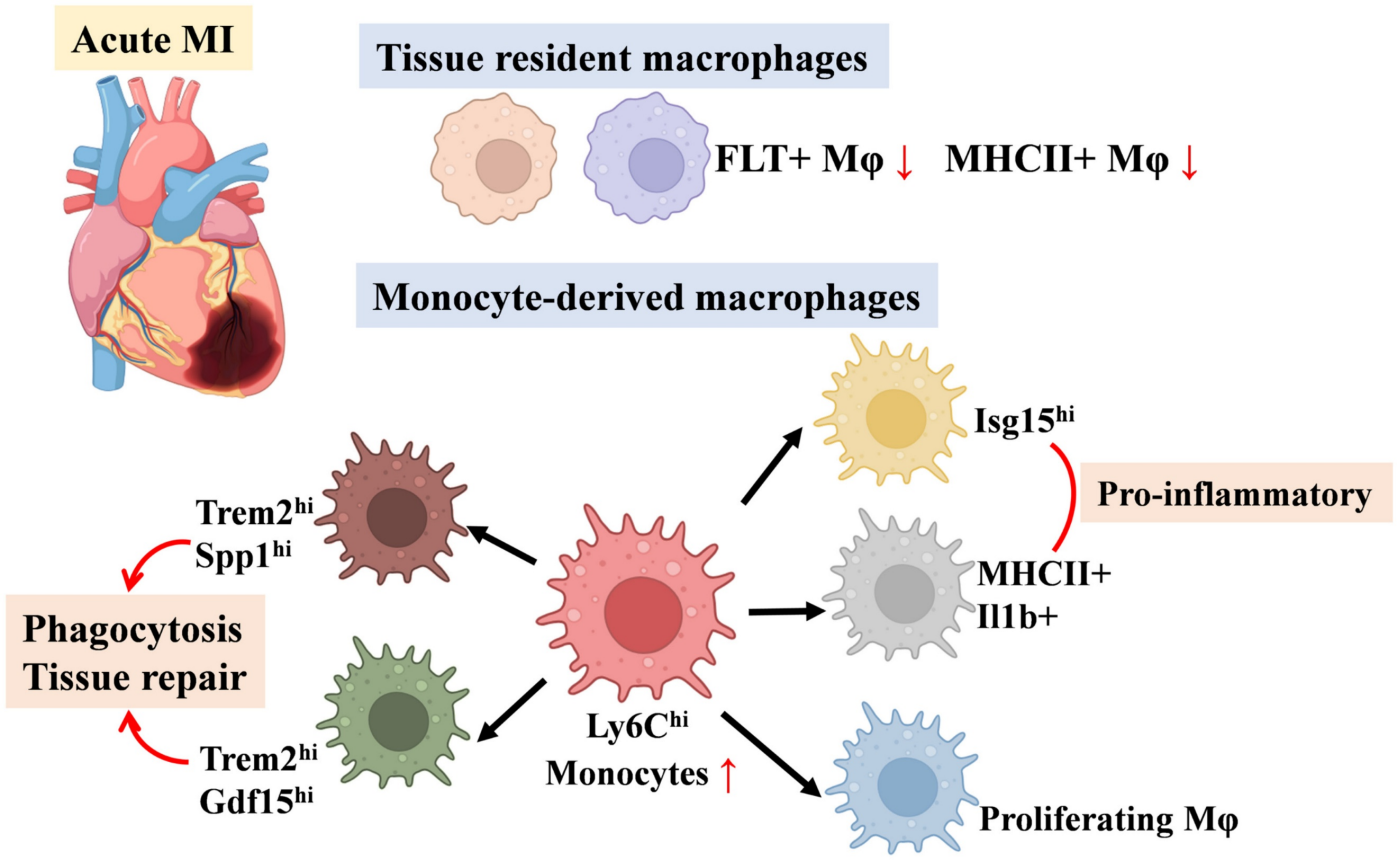
Schematic of Changes in Major Macrophage Subsets in Myocardial Infarction.

In summary, these studies highlight the dynamic shifts in resident and monocyte-derived macrophage populations across cardiac regions following MI—including the ischemic zone (comprising the infarct and peri-infarct regions) and remote non-infarcted myocardium—and their distinct contributions to tissue repair and remodeling (Figure 1). We have compiled macrophage subpopulations, their markers, and functional characteristics from MI-related studies as a quick reference for readers (Table 1).

### Atherosclerosis

Atherosclerosis is a chronic, lipid-driven vascular inflammatory disease characterized by the formation of plaques in large arteries, driven primarily by the subendothelial deposition of lipoproteins in regions of disturbed blood flow 89. Deposited lipoproteins generate pro-inflammatory derivatives, recruit leukocytes 90, and drive the polarization of resident immune cells 91. The balance between pro-inflammatory and inflammation-resolving processes within plaques determines whether lesions remain stable or regressive, or instead progress toward instability and rupture 92. Macrophages, as key mediators of the inflammatory response, play critical roles throughout all stages of atherosclerosis, including plaque initiation, calcification, rupture, and regression. They accumulate through both the recruitment of circulating monocytes and the proliferation of locally differentiated macrophages. During the progression of atherosclerosis, macrophages produce a broad array of pro-inflammatory and anti-inflammatory mediators, pro-thrombotic tissue factors, and proteolytic enzymes. These secreted factors modulate the growth, cellular composition, and stability of atherosclerotic plaques, thereby significantly influencing disease outcomes 93-95.

#### Metabolic changes of macrophages in atherosclerosis

Previous studies have demonstrated that macrophages within atherosclerotic plaques undergo profound metabolic reprogramming 96. These macrophages exhibit increased glucose uptake and enhanced glycolytic activity, likely mediated by upregulation of the HIF1α-GLUT1 axis, which promotes a pro-inflammatory phenotype 32. Despite the abundance of fatty acids and cholesterol in plaques—which could theoretically support oxidative phosphorylation—mitochondrial dysfunction limits oxygen consumption. The accumulation of fatty acids and cholesterol instead activates TLR4 signaling, further exacerbating inflammation 97. In contrast, during the regression phase of plaques, macrophages may shift to lipid-based metabolism under the influence of pro-resolving mediators or efferocytosis, thereby facilitating the resolution of inflammation and promoting tissue repair 98.

#### Resident macrophages in atherosclerosis

Resident macrophages are present in both healthy and atherosclerotic aortic adventitia. In healthy aortas, these macrophages express higher levels of Lyve1, whereas atherosclerotic aortas show increased Ccr2 expression 99. Atherosclerotic aortas contain both true resident macrophages and newly recruited cells, which may acquire similar gene expression profiles upon infiltration. Notably, resident macrophages in atherosclerosis correspond to TLF+ macrophages. Depletion of Lyve1+ macrophages in Lyve^wt/cre^ Csf1r^flox/flox^ mice leads to increased arterial stiffness and collagen deposition, underscoring their essential role in maintaining vascular homeostasis 100. Pathway analyses indicate that resident-like plaque macrophages are actively involved in receptor-mediated endocytosis 101,102.

Additionally, the aortic intima contains a distinct population of resident macrophages, termed aortic intimal resident macrophages (Mac^AIR^) 103. These cells originate from bone marrow progenitors that colonize the aorta at birth and maintain their population through self-renewal. Although transcriptionally similar to foam/Trem2^hi^ macrophages, Mac^AIR^ cells display higher expression of MHCII transcripts and lower levels of foam cell-associated genes (Trem2, Spp1). Mac^AIR^ cells act as primary precursors of foam cells during early atherosclerosis, mediating initial lipid deposition via SR-A1/CD36-mediated uptake of oxidized (oxLDL) and aggregated LDL (agLDL). Their tissue-resident nature enables lipid accumulation before monocyte infiltration, and elevated baseline expression of lipid metabolism genes (e.g., CD36) primes them for rapid lipid uptake during hyperlipidemia. Brief cholesterol elevation amplifies both lipid loading and Mac^AIR^ proliferation, accelerating fatty streak formation. As atherosclerotic plaques advance, the limited proliferative capacity of Mac^AIR^ cells, combined with persistent hypercholesterolemia, overwhelms their cholesterol metabolic potential, promoting accelerated foam cell differentiation and apoptosis. Consequently, Mac^AIR^ populations peak during early lesion development but decline as monocyte-derived macrophages predominate in advanced plaques 104.

#### Pro-inflammatory macrophages and TREM2^hi^ / foam macrophages in atherosclerosis

The intima of atherosclerotic aortas, compared to healthy arteries, contains distinctive macrophage populations, including pro-inflammatory and TREM2^hi^ macrophages. Pro-inflammatory macrophages predominantly localize to the shoulder regions of plaques and are defined by high expression of pro-inflammatory chemokines, Tlr2, and Nlrp3^105^. Zernecke et al. further identified a population, termed “inflammatory-Mφ,” comprising two subsets 105: inflammatory-Nlrp3 macrophages, which highly express Nlrp3 and Il1b, and CCR2^int^ MHCII+ macrophages, which exhibit intermediate expression of Ccr2 and express MHCII-related transcripts such as Cd74, H2-Aa, and H2-Eb1. Inflammatory macrophages constitute the dominant macrophage population of the plaque intima, and as the principal non-foam cells exclusive to atherosclerotic aortas, act as key drivers of inflammation in advanced lesions. Kim et al. demonstrated that non-foam macrophages display even greater pro-inflammatory activity than foam macrophages, underscoring their central role in promoting plaque inflammation 101.

Recent research has redefined Trem2hi macrophages as lipid-laden foam cells, refining the traditional foam cell classification. Single-cell sequencing consistently shows that Trem2^hi^ macrophages possess classic foam cell features 101. These cells express high levels of Trem2, CD9, Fabp4, Apoe, and Apoc1, and are primarily localized within plaque intima and necrotic cores 99. Zernecke et al. further identified two Trem2hi/foam macrophage subsets: the Trem2^hi^-Slamf9 subset, with high expression of Slamf9, Ch25h, Cd72, and related markers, and the Trem2^hi^-Gpnmb subset, which is marked by elevated Gpnmb, Atp6v0d2, other foam cell-associated transcripts, and Trem2-related genes such as Lpl, Lipa, Fabp5, Apoc1, and Apoe 105. Pathway analyses reveal that Trem2^hi^ foam macrophages are enriched for processes including organic substance metabolism, lipid metabolism, cholesterol efflux regulation, and oxidative stress pathways 101,102. Van Kuijk et al. demonstrated that Trem2^hi^ macrophages also highly express SPP1 and MMPs, indicating a role in promoting fibrosis, mirroring the fibrotic activity of Trem2+ hepatic macrophages 106. These findings highlight the metabolic and functional specialization of Trem2^hi^ macrophages within atherosclerotic plaques.

#### Other Macrophage Subtypes in Atherosclerosis

Atherosclerosis additionally involves several distinct macrophage subtypes, including proliferative macrophages, IFNICs, and intimal macrophages. Proliferative macrophages are defined by high expression of Birc5, and display elevated levels of the proliferation marker Mki67 107. Van Kuijk et al. 106 and Zernecke et al. 105 reported the presence of numerous IFNIC macrophages, while Cai et al. 108 identified interferon-responsive macrophages. However, it remains unclear whether interferon-activated macrophages represent a homeostatic population or arise exclusively in disease, although their association with type I interferon signaling suggests a pro-atherosclerotic role. Additionally, Zernecke et al. identified a macrophage subset resembling intimal macrophages in the aortas of atherosclerotic mice. These cells share a gene expression profile with subadventitial macrophages (CD226+CD11c+MHCII+) found in the SPM/cavity regions 109. The origin and precise functions of these macrophages, which have not been previously identified in the aorta, remain uncertain and merit further investigation through single-cell analyses.

#### Human Atherosclerotic Plaque Macrophage Clusters

In analyses of human atherosclerotic plaques, Zernecke et al. identified three primary macrophage clusters: hInflammatory-Mφ, hFoamy/TREM2^hi^-Mφ, and hLYVE1-Mφ, along with smaller clusters such as type I interferon response macrophages (hIFNIC-Mφ) and proliferating macrophages (hProlif cluster) 105. These findings closely parallel the macrophage subsets observed in murine atherosclerosis models. Expression of signature markers for major vascular macrophage subtypes demonstrates a conserved distribution of cell clusters across species. Winkels et al. identified two potential human macrophage subtypes 110: CD11b+HLA-DR^med^ and CD11b+CD36+. Similarly, Depuydt et, al. 111 and Fernandez et, al. 112 reported the presence of both pro-inflammatory and anti-inflammatory macrophage clusters in human plaques. Of note, human foam macrophages display relatively stronger anti-inflammatory properties compared to non-foam macrophages, as they significantly suppress Il1b expression, implicating their potential role in modulating inflammatory responses within plaques.

**Figure 2:**
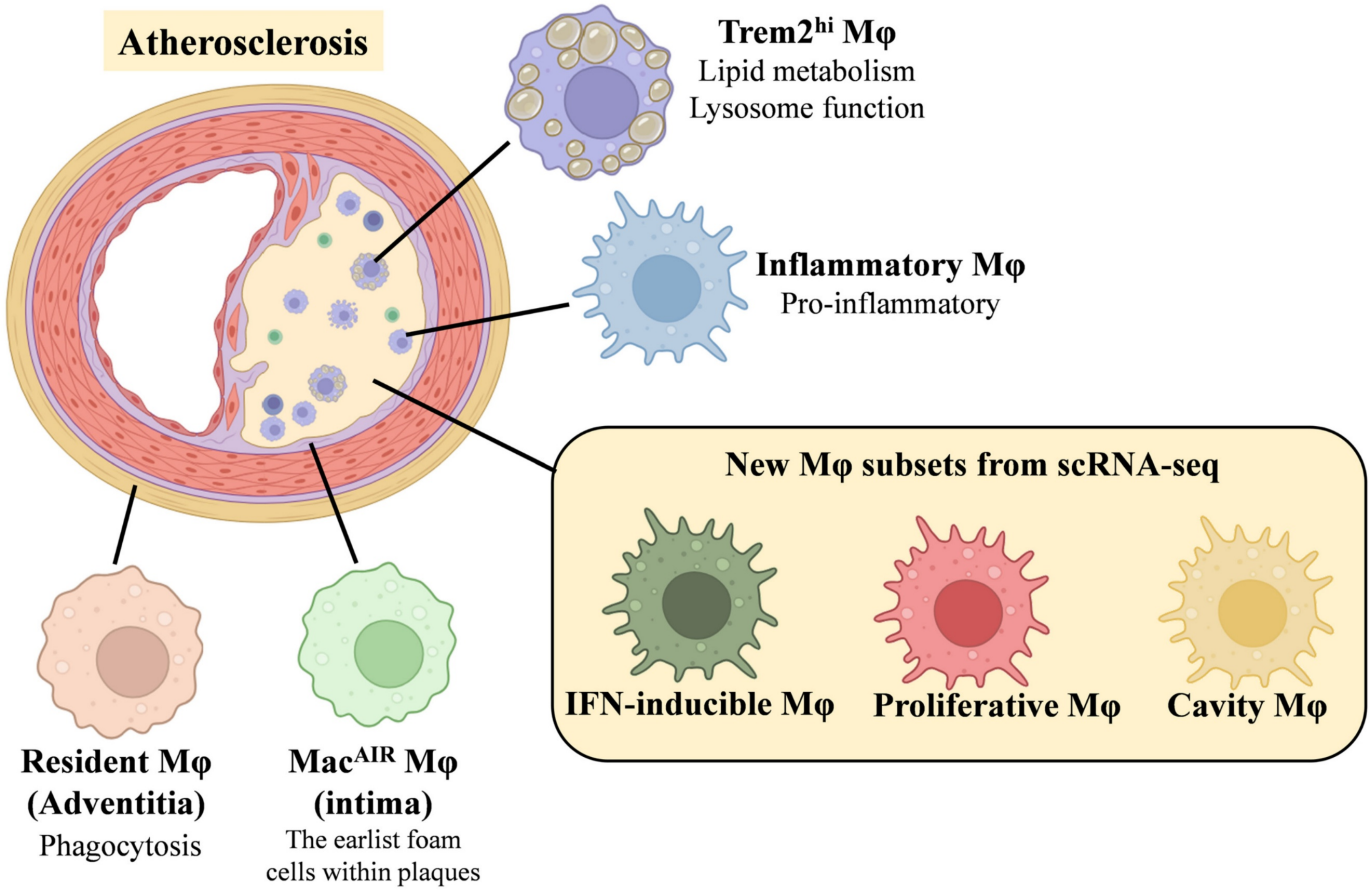
Schematic of Changes in Major Macrophage Subsets in Atherosclerosis.

In conclusion, the distribution of macrophage subsets involved in these atherosclerosis studies is shown in Figure 2. These macrophage subsets, their markers and functional characteristics are shown in Table 2.

### Heart Failure Induced by Non-Ischemic Cardiomyopathy

Heart failure (HF) is a global health challenge, affecting over 23 million individuals and imposing a substantial burden on healthcare systems 113,114. Cardiac overload triggers the release of pro-inflammatory cytokines, which drive monocyte infiltration into the myocardium—a critical component of the immune response and a major factor in disease progression 115,116. In non-ischemic conditions, diverse stimuli can activate fibrotic signaling pathways in macrophages, leading to myocardial fibrosis. For instance, pressure overload (PO) induces fibrosis through mechanical stress and activation of the renin-angiotensin-aldosterone system (RAAS), whereas reactive oxygen species (ROS) play a central role in the pathogenesis of dilated cardiomyopathy (DCM). Interstitial fibrosis in non-ischemic heart injury represents a chronic and progressive process, predominantly driven by sustained, unregulated inflammation and persistent activation of profibrotic pathways. As cardiac injury advances, the heart loses its capacity to manage normal volume and/or pressure loads. The resulting microenvironment of the failing heart is characterized by extracellular matrix accumulation, impaired microcirculation, and excessive activation of immune cells, all of which further exacerbate HF progression 117.

#### Dynamic changes of macrophages in HF

Non-ischemic cardiomyopathy (NICM) animal models can be generated using various stressors, such as transverse aortic constriction (TAC) or angiotensin II (Ang-II) infusion. These models display characteristic pathological features, including left ventricular dysfunction, progressive interstitial fibrosis, and deteriorating cardiac function. In the Ang-II infusion-induced HF model, macrophage accumulation within the heart peaks at day 7, a pattern that mirrors the dynamic changes observed in the TAC-induced mouse HF model. After 7 days of Ang-II infusion, macrophage populations present in the steady-state heart—particularly those associated with reparative functions, such as the Timd4 and AP-1 clusters—show significant reductions in number. In contrast, pro-inflammatory macrophage subsets, including those with increased ribosome synthesis (stem cell-like) and interferon-stimulated gene (ISG)-related clusters, exhibit pronounced expansion 118,119.

In the TAC-induced HF mouse model, both resident macrophages (Timd4+ Ccr2-) and monocyte-derived macrophages (MoMF, Ccr2+) increase by 7 days post-TAC. By 28 days post-TAC, the numbers of most macrophage subsets return to baseline 118. At both 7 and 28 days after TAC, the numbers of recruited M1-like CCR2+ Il1b+ macrophages are significantly elevated compared to sham controls. Resident macrophages, including CCR2- MHCII^lo^ M1-like and CCR2- MHCII^hi^ M2-like cells, also show modest increases at these time points. Martini et al. proposed that CCR2- MHCII^hi^ M2-like macrophages contribute to immune surveillance by promoting tissue repair and antigen presentation, whereas CCR2- MHCII^lo^ M1-like cells maintain homeostasis through phagocytosis of dead cardiomyocytes 118. Conversely, the M1-like Ccr2+ Osm+ Il1b+ subset exerts pro-inflammatory effects through expression of oncostatin M (Osm), a cytokine linked to organ dysfunction 118.

Treatment with a monoclonal α-CD115 antibody, which preferentially depletes resident macrophages, was found to exacerbate fibrosis and heart failure in TAC mice 120. These results suggest that resident macrophages are essential for attenuating cardiac deterioration and preventing fibrosis during early cardiac remodeling. In contrast, MoMFs exerted profibrotic effects in Ccr2 knockout mice. Consistent with these findings, pressure overload induced significant interactions between pro-inflammatory M1-like macrophages and activated Postn+ fibroblast subsets. CD72^hi^ cardiac macrophages have been identified as a pro-inflammatory subset implicated in inflammation and cardiac injury following myocardial infarction 121. This finding indicates that CD72 can serve as a marker for infiltrating monocytes/macrophages in TAC-induced heart failure. Cd72 expression, along with Ccr2, demonstrates strong similarity along the pseudo-time trajectory, and Cd72 is also highly expressed in ISG-related clusters, further linking it to pro-inflammatory Ccr2^hi^ monocytes/macrophages. Clinically, heart failure patients with DCM exhibit increased levels of CD72^hi^ macrophages, suggesting that the abundance of inflammatory macrophages serves as a negative predictor of cardiac recovery 121.

#### Differential Functions of Macrophage Subsets in Human Heart Failure

Using scRNA-seq, Rao et al. classified human HF macrophages into three principal subsets: CCR2-HLA-DR^hi^, CCR2+HLA-DR^hi^, and TREM2+ macrophages, which correspond to murine Ccr2-MHCII^hi^, Ccr2+MHCII^hi^, and Trem2+ subsets, respectively 122. In diseased human hearts, CCR2-HLA-DR^hi^ macrophages are more prevalent in the right ventricle (RV) of DCM patients, whereas CCR2+HLA-DR^hi^ macrophages dominate in the left ventricle (LV), which is more severely fibrotic. The CCR2+HLA-DR^hi^ subset displays pro-inflammatory activity, consistent with previous reports associating increased CCR2+ monocyte-derived macrophages with poor prognosis in heart failure. The specific expression of SPP1 and LGALS3 in TREM2+ macrophages suggests a potential role in promoting angiogenesis and immune suppression, contributing to protective responses under stress. Another study of human heart failure demonstrated divergent roles for monocyte-derived and resident cardiac macrophages in the disease process 123. Monocyte-derived macrophages are enriched in hearts of patients who do not recover after left atrial volume reduction surgery (LAVD), expressing pro-inflammatory genes such as PLAUR, IL1B, TNF, and CCL4, which are associated with myocarditis and heart failure. These results emphasize that inflammatory macrophages serve as negative prognostic indicators for cardiac recovery. In contrast, CD163+ resident cardiac macrophages, which are significantly depleted during heart failure, return to normal levels in patients who achieve recovery. These macrophages exhibit transcriptional profiles closely linked to cardiac repair and remodeling, indicating their key regulatory role in heart failure recovery.

**Figure 3:**
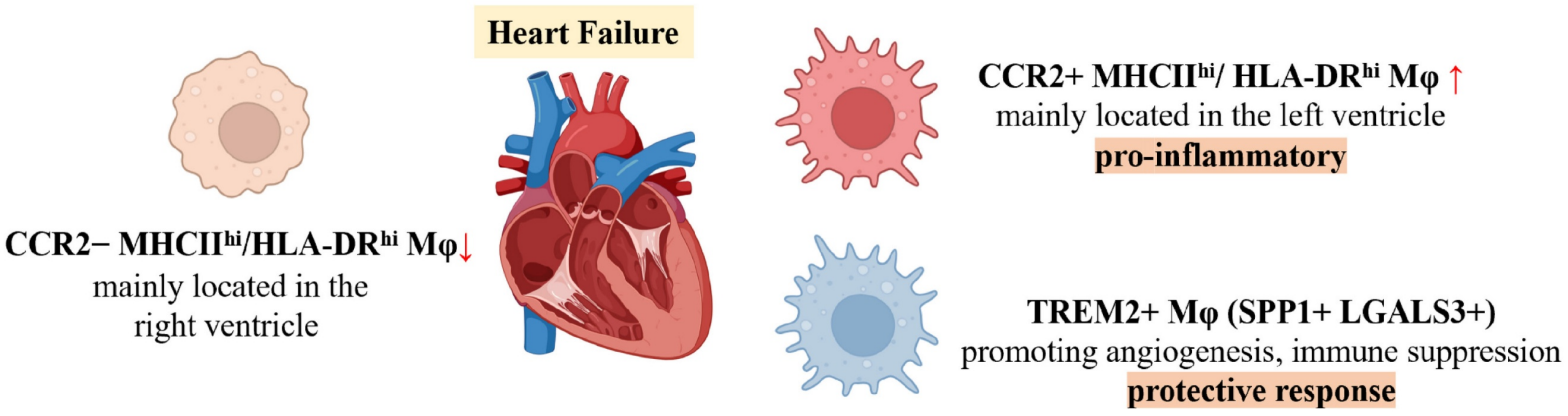
Schematic of Changes in Major Macrophage Subsets in Heart failure.

In conclusion, Figure 3 illustrates the macrophage subsets involved in heart failure, while Table 3 provides a detailed overview of these macrophage subsets, including their markers and functional characteristics.

### Myocarditis

Myocarditis is characterized by dysregulated cardiac inflammation driven by dynamic macrophage heterogeneity 124. In experimental models, cardiac macrophage expansion predominantly results from the recruitment of Ly6C^hi^CCR2+ monocytes, which differentiate into MHC-II^hi^CCR2+ macrophages during the early stages of injury 125-127. Using the experimental autoimmune myocarditis (EAM) model, Hua et al. 128 demonstrated that the major macrophage population present during the acute phase—distinguished by differential expression of Nos2, Arg1, and Ass1—produces nitric oxide through Ass1-mediated biosynthesis, thereby enhancing phagosomal antigen processing, IFN-γ responsiveness, and ROS metabolism. During the subacute inflammatory phase, a separate macrophage subset marked by high Ccl8 expression facilitates the processing and presentation of foreign peptide antigens via MHCII, and mediates cellular chemotaxis and monocyte migration. In the chronic myopathic phase, macrophage clusters with distinct expression of Tnf, Il-10, Vsig4, and Tnip3 contribute to the activation of mitogen-activated protein kinase (MAPK) cascades, TNF signaling, and NF-κB pathways, which collectively attenuate inflammation and promote wound healing through the synergistic effects of Il-10 and Tnip3. In giant cell myocarditis (GCM), multiple macrophage clusters have been identified 129, including monocyte-derived populations as well as mixed clusters containing both monocyte-derived and resident macrophages. Monocyte-derived macrophages express elevated levels of Prdx1 and Prdx5, genes associated with autoimmunity, while mixed clusters express members of the Ms4a gene family; other clusters express Nr4a1 and Pf4. Nr4a1 acts to inhibit macrophage polarization towards the pro-inflammatory M1 phenotype, whereas Pf4, a broad-spectrum inflammatory chemokine, functions to restrain the activation of resident macrophages.

The increasing use of immune checkpoint inhibitors (ICIs), including PD-1/PD-L1 and CTLA-4 inhibitors, in cancer therapy has brought greater attention to immune-related adverse events, among which myocarditis is notably severe 130. In genetic models of ICI-induced myocarditis, a CCR2+ monocyte-derived macrophage subset characterized by Cxcl9+Cxcl10+ expression exhibits an activated phenotype and exacerbates disease progression via three principal mechanisms: (1) T-cell hyperactivation through CXCL16/CXCR6-mediated CD8+ T cell interactions and CXCL9/CXCL10-CXCR3-dependent recruitment and activation of both CD4+ and CD8+ T cells, which amplify cytotoxic attacks on myocardial cells; (2) amplification of chemokine storms via CCL2/MCP1 and CCL7/MCP3 production, recruiting peripheral immune cells and intensifying myocardial inflammation; and (3) direct myocardial injury mediated by effector T-cell activation, antibody-dependent cellular cytotoxicity (ADCC), and phagocytosis. This pathogenic cascade is orchestrated by IFN-γ-STAT1 signaling, and therapeutic inhibition of the JAK2/STAT1 axis with ruxolitinib has been shown to reduce cardiovascular mortality in patients with ICI-induced myocarditis. In comparison to Cxcl9+Cxcl10+ macrophages, Nlrp3+ macrophages are enriched for genes involved in responses to LPS, regulation of IL-1β production, and stromal cell proliferation. CD163+ resident macrophages are enriched for pathways that regulate epithelial cell proliferation and stress response.

These findings highlight the diverse roles of macrophage subpopulations in myocarditis (Table 4).

### Diabetic Cardiomyopathy

Diabetic cardiomyopathy (DbCM) is defined by structural and functional abnormalities of the myocardium in diabetic patients, with metabolic dysregulation and myocardial fibrosis as hallmark features 133,134. During early hyperglycemia, cardiac interstitial macrophage infiltration is triggered by advanced glycation end-product (AGE) accumulation, adipokine secretion, activation of the RAAS, microvascular dysfunction, and increased oxidative stress. The progression of DbCM is primarily driven by impaired insulin signaling and mitochondrial dysfunction 135, both of which disrupt oxidative phosphorylation in cardiomyocytes. Insulin resistance hampers glucose uptake via GLUT1 and GLUT4 and inhibits fatty acid oxidation by suppressing key rate-limiting enzymes 136. The subsequent mismatch between mitochondrial dysfunction and excessive fatty acid accumulation results in elevated ROS production, which further skews macrophages toward a pro-inflammatory CCR2+ phenotype. Dectin-1, a receptor predominantly expressed by macrophage pattern-recognition receptors (PRRs), promotes this inflammatory polarization in hyperglycemic environments by activating the Syk/NF-κB pathway 137. These pro-inflammatory macrophages secrete cytokines including TNF-α, IL-1β, and IL-6, which markedly increase the expression of resistin—an adipokine that exacerbates insulin resistance—thereby further perpetuating hyperglycemia. Moreover, resistin itself can enhance the production of inflammatory cytokines, creating a self-reinforcing vicious cycle.

Cardiac fibrosis—a defining feature of mid- to late-stage DbCM—results from macrophage-fibroblast interactions that drive interstitial and perivascular fibrosis 133,139. In mouse models of DbCM, endothelial cell and macrophage numbers decrease while fibroblast and cardiomyocyte populations increase, reflecting aggravated fibrosis and endothelial injury 140. In diabetic mouse hearts induced by HFD/STZ, Egfr and Pdgfra are highly expressed in cardiac fibroblasts, whereas macrophages exhibit increased Pdgfc expression 141. These data indicate that macrophage-fibroblast crosstalk contributes to the development of myocardial fibrosis in DbCM. Notably, dynamic changes in macrophage subsets over time during DbCM progression remain understudied. Future research should employ longitudinal multi-omics and macrophage lineage tracing strategies to comprehensively characterize the evolution of macrophage phenotypes and functions, thereby providing a robust theoretical framework for developing targeted interventions in DbCM.

### Cardiac Sarcoidosis

**Figure 4:**
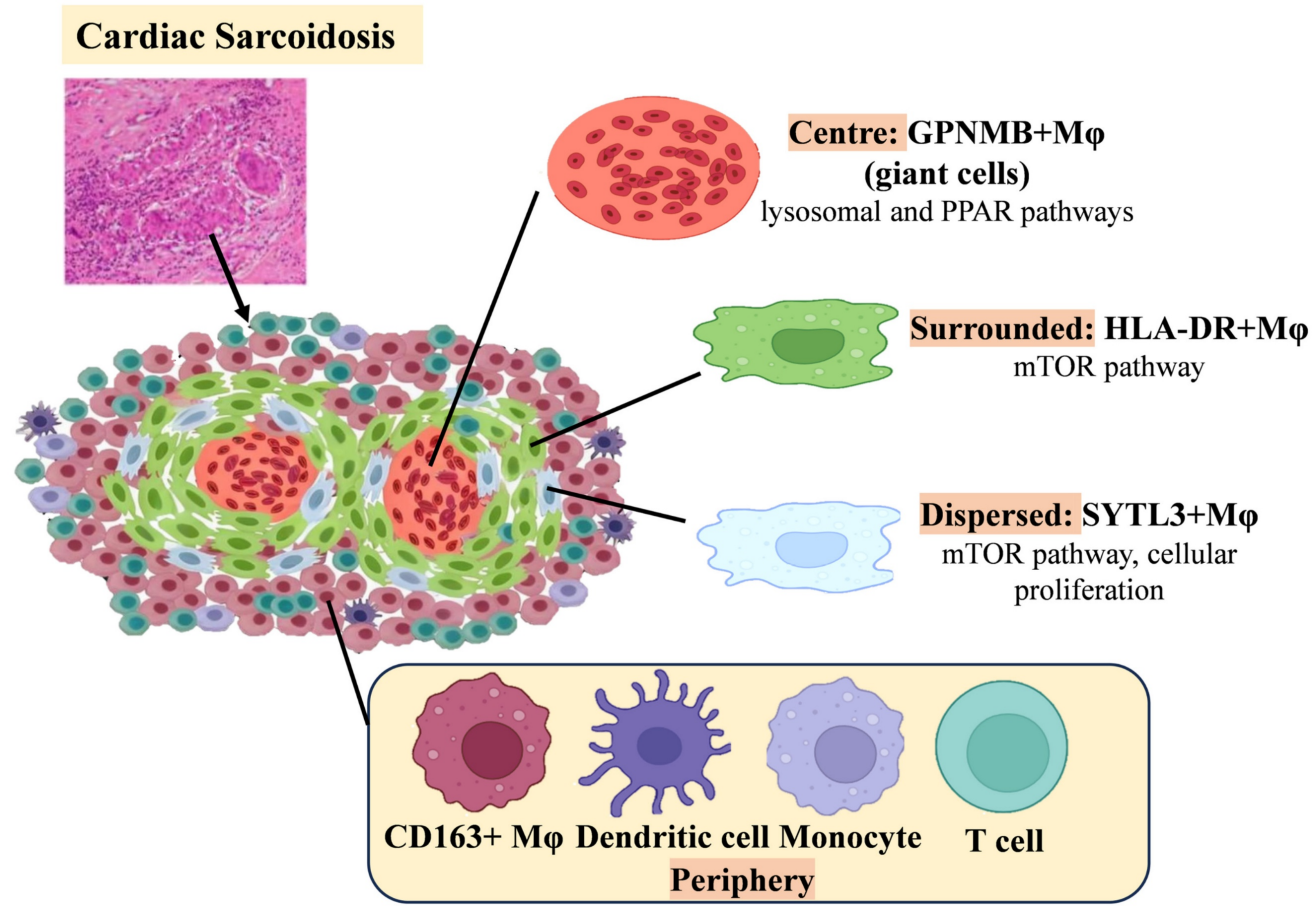
Schematic of Changes in Major Macrophage Subsets in Cardiac Saroidosis.

Cardiac sarcoidosis (CS) is histologically defined by granulomatous inflammation, with granulomas composed primarily of macrophages 142. One of the main diagnostic challenges in CS is distinguishing it from other inflammatory cardiomyopathies—such as giant cell myocarditis (GCM) and lymphocytic myocarditis—due to overlapping histopathologic features. Recent spatial transcriptomic analyses have elucidated distinct cellular architectures that facilitate this differentiation 132. Notably, GPNMB+ (glycoprotein non-metastatic melanoma protein B) multinucleated giant cells are surrounded by dense infiltrates of HLA-DRhi macrophages in CS, forming a distinctive granulomatous structure absent in other types of inflammatory heart disease. Within these granulomas, SYTL3+ macrophages are diffusely distributed, whereas CD163+ macrophages, CD1c+ dendritic cells, non-classical monocytes, and T cells localize to the periphery and external regions (Figure 4). Transcriptomic profiling reveals unique enrichment of lysosomal and PPAR signaling pathways in GPNMB+ giant cells, along with selective mTOR pathway activation—a key regulator of cellular proliferation—in HLA-DR^hi^ and SYTL3+ macrophages. SYTL3+ macrophages may represent a transitional differentiation stage, potentially giving rise to both GPNMB+ giant cells and adjacent mature epithelioid-like histiocytes (HLA-DR^hi^ macrophages).

Subsequent studies have identified GPNMB as a novel marker of multinucleated giant cells in CS, with its regulation potentially mediated by the microphthalmia-associated transcription factor (MITF) family. Although GPNMB immunohistochemistry detects giant cells in both CS and GCM, the spatial organization of HLA-DR^hi^ macrophages offers a means of diagnostic distinction: in CS, GPNMB+ giant cells are closely encircled by HLA-DR^hi^ macrophages, whereas in GCM, these cell populations are more diffusely distributed throughout lesions. This organizational distinction provides a practical diagnostic tool. Combining GPNMB staining with HLA-DR spatial mapping thus enhances diagnostic accuracy, addressing longstanding challenges in distinguishing inflammatory cardiomyopathies. These findings underscore the value of multi-omics technologies in refining histopathological criteria.

## Discussion

### Decoding Macrophage Heterogeneity through scRNA-Seq

scRNA-seq has transformed our understanding of macrophage heterogeneity in cardiovascular pathology by revealing distinct functional subsets shaped by disease-specific microenvironments. Monocyte-derived CCR2+ macrophages initiate inflammatory cascades and promote adverse ventricular remodeling, whereas resident macrophage populations confer cardioprotective effects that maintain cardiac homeostasis and functional integrity 143,144. These broad categories further differentiate into context-dependent subpopulations (Table 5): for example, TREM2^hi^ foam cells in atherosclerotic plaques facilitate lipid uptake and enhance cholesterol efflux; post-MI TREM2^hi^ macrophages upregulate Arg1 and IL-10 to promote reparative processes; SPP1+ macrophages secrete TGF-β and IL-10, exacerbating fibrosis; and CD72^hi^ subsets amplify inflammatory signaling, accelerating heart failure progression. These populations are defined not only by unique surface markers, but also by specific metabolic pathways and epigenetic regulators, making them compelling therapeutic targets. Importantly, global depletion of cardiac macrophages may impair left ventricular remodeling due to the loss of resident macrophage subsets critical for myocardial homeostasis 145,146. Therefore, selective targeting of specific macrophage subsets is essential and requires a comprehensive understanding of their functional diversity within mixed populations. In pathological settings, the inflammatory microenvironment can shift cardiac macrophages from reparative to detrimental phenotypes, meaning treatment efficacy varies depending on the timing and subsets targeted 147. Integrating scRNA-seq with other omics technologies enables multimodal analysis of macrophage interactions with cardiomyocytes, fibroblasts, and other immune cells, helping to resolve niche-specific activation trajectories.

### New strategies for targeted macrophage therapy

Current therapeutic approaches targeting specific macrophage subtypes remain imprecise, typically focusing on M2-like macrophages and their associated anti-inflammatory mediators. Emerging strategies include: (1) Direct targeting of subset-specific receptors—soluble TREM2 can reprogram post-MI macrophages toward reparative phenotypes, and CCR2 antagonists (e.g., cenicriviroc) reduce inflammatory monocyte infiltration in atherosclerosis and MI; (2) Metabolic reprogramming—inhibition of glycolysis promotes oxidative metabolism and tissue repair, while PPARγ agonists enhance fatty acid oxidation and cholesterol efflux; (3) Epigenetic engineering—CRISPR-Cas9-mediated editing of genes such as TREM2 or BHLHE41 in foam cells modulates lipid metabolism and fibrotic pathways 148; (4) Nano-targeted delivery—nanoparticle platforms selectively deliver drugs or siRNA to Lyve1+ resident macrophages or Ly6C^hi^ monocytes, thereby preserving beneficial subsets 149; (5) Integration of AI-driven multi-omics analyses and deep-learning-based screening facilitates the personalized design of macrophage-targeted therapies, with the potential to improve cardiovascular outcomes.

### Spatial Transcriptomics Reveals Macrophage Niche in Cardiovascular Disease

Spatial transcriptomics, an emerging technique, complements scRNA-seq by retaining the spatial context of gene expression within tissues. This approach is particularly valuable in cardiovascular research, as it enables the mapping of macrophage localization in relation to other cardiac and vascular cell types, offering new insights into the tissue microenvironment that regulates macrophage function. Tissue homeostasis and cell function rely on direct interactions between neighboring cell types; spatial omics methods thus provide crucial information about how distinct macrophage subpopulations interact with their cellular milieu to coordinate tissue responses. For example, following myocardial infarction, different regions (control, peri-infarct, remote myocardium) exhibit spatially distinct enrichment of macrophage functional states, providing clarity on how cell states are modulated by their local neighborhoods. Integration of single-cell multi-omics with spatial data has uncovered new relationships between macrophage subtypes and myofibroblast differentiation during cardiac remodeling. Notably, it is important to recognize that macrophage markers identified from nuclei in human tissue differ substantially from those acquired via whole-cell sequencing. Therefore, macrophage subpopulations defined through nuclear sequencing often cannot be mapped directly onto conventional classifications, underscoring the need for more refined classification strategies in human studies.

### Technical Bottlenecks in Cardiac Macrophage Metabolomics

While metabolomics has seen widespread application in macrophage research, particularly in oncology, its use in cardiovascular macrophages remains limited. Cancer studies have elucidated that tumor cells reprogram macrophage metabolism by releasing metabolites such as succinate and fatty acids into the microenvironment 150,151. By contrast, most insights into cardiovascular macrophage metabolism are derived from transcriptomic profiling and knockout mouse models 32,152. Since metabolic activity is regulated at multiple levels, transcriptomic data alone are insufficient to fully explain metabolic phenotypes. Although unbiased metabolomics provides critical functional insights, several technical barriers hinder its adoption. Cardiac macrophage studies face unique challenges: (1) Their low abundance (representing only ~7% of cardiac cells 153) complicates isolation, risking tissue-level metabolome analyses that misrepresent macrophage-specific metabolic states; (2) Tissue dissociation can induce metabolic artifacts; (3) High-throughput platforms (MS/NMR) often require trade-offs between quantitative accuracy and coverage, necessitating resource-intensive targeted validation; (4) Incomplete metabolite annotation limits functional interpretation 154. As a result, most studies rely on more readily accessible primary macrophages (e.g., BMDMs, peritoneal macrophages) rather than tissue-resident cardiac populations 34. For instance, Tabas et al. identified apoptotic cell-derived arginine as essential for efferocytosis-induced macrophage polarization using BMDMs 155, while Thorp's group demonstrated that engulfed fatty acids drive pro-resolving functions in peritoneal macrophages through mitochondrial metabolism 19. However, these observations may not accurately capture the metabolic dynamics of cardiac macrophages. A practical approach often involves *in vitro* screening of metabolic pathways, with subsequent *in vivo* validation. Nonetheless, such reductions risk oversimplifying the complex interplay between cardiac macrophages and their tissue niches. Future methodological advances should address the need for increased sensitivity (enabling single-cell metabolomics) and spatial resolution, while minimizing *ex vivo* manipulation artifacts.

### The limitations of Multi - Omics

Current multi-omics approaches in cardiac macrophage research are hampered by limitations in temporal and spatial resolution, incomplete metabolome coverage, and batch effects, all of which impede mechanistic insights and translational advances. Firstly, static omics methods (like scRNA-seq) cannot adequately capture the temporal dynamics of macrophage phenotypic transitions—for example, delineating the shift from pro-inflammatory to reparative states following MI requires time-resolved analysis, which is lacking in snapshot datasets. Longitudinal multi-omics paired with lineage-tracing systems (such as Cre/Dre recombinases) offer promising solutions. Secondly, spatial transcriptomics typically aggregates multiple cells per spot, obscuring detailed macrophage-microenvironment interactions; achieving true single-cell resolution remains a priority. Furthermore, integration of distinct omics layers is complicated by platform-specific biases and batch effects, necessitating advanced computational harmonization—such as the application of multimodal variational autoencoders—to reliably elucidate cross-omic relationships 156. Lastly, metabolomics is constrained by limited MS and NMR sensitivity, particularly for detecting atherosclerosis-relevant lipids critical to foam cell formation. Although emerging techniques like MALDI-MSI enable single-cell metabolic imaging 157, widespread adoption is limited by technical immaturity.

### Future Directions

#### Advancements and Challenges in Single-Cell Multi-Omics

Single-cell technologies now enable multidimensional analysis of cellular processes, although integration across omics layers remains challenging. Traditional CRISPR-based screens (e.g., Perturb-seq combined with scRNA-seq) elucidate transcriptomic responses to genetic perturbations but lack spatial context. Conversely, spatial transcriptomic platforms such as multiplexed error-robust fluorescence *in situ* hybridization (MERFISH) provide precise tissue architecture but are generally incompatible with CRISPR perturbations 158. Perturb-FISH bridges this gap, enabling simultaneous mapping of genetic perturbations, transcriptomes, and spatial information at single-cell resolution 159. Validated in THP1 macrophages, this approach holds promise for dissecting macrophage heterogeneity in cardiovascular tissues. Additionally, spatial single-cell proteomics platforms (e.g., PhenoCycler-Fusion) provide visualization and quantification of protein expression at single-cell resolution, furnishing critical spatial context for understanding cellular localization and function 160. Spatial metabolomics using imaging mass cytometry (IMC) and mass spectrometry imaging (MSI) is also increasingly employed to identify cellular biomarkers and metabolites while preserving tissue architecture. Despite these advances, single-cell metabolic imaging remains technically underdeveloped. Recent multimodal MSI workflows integrating MALDI-MSI and IMC enable single-cell analysis of metabolic heterogeneity and its relationship to specific cell populations in human tissue 161, though scalability, reproducibility, and efficiency require further validation before routine adoption. Collectively, these innovations underscore the transformative potential of single-cell multi-omics approaches, while highlighting the need for continued technical refinement to achieve seamless integration across molecular layers.

#### AI-Driven Multi-Omics Integration in Cardiac Macrophages

The complexity of multi-omics datasets spanning transcriptomic, epigenetic, metabolic, and spatial dimensions challenges conventional data integration approaches. Artificial intelligence (AI) and machine learning (ML) mitigate these challenges in four principal domains 162. First, for data integration and analysis, AI/ML models—including supervised, unsupervised, and deep learning methods—facilitate harmonization across multi-omics datasets. Algorithms such as random forests, support vector machines, and transformer architectures excel at identifying cross-omic patterns and relationships often missed by standard statistical techniques. Initiatives like AtheroNET exemplify how such integration is fundamental for elucidating macrophage-driven mechanisms in cardiovascular pathology. Second, in biomarker discovery, AI/ML accelerates the identification of novel macrophage-specific markers by mining integrated multi-omics profiles. Third, in mechanistic insight, these tools help to unravel complex biological networks, clarifying macrophage-mediated pathways (e.g., inflammation, oxidative stress, mitochondrial dysfunction) underlying cardiac disease progression. Finally, in predictive modeling, ML frameworks combine multi-omics and clinical data to forecast disease progression and cardiovascular outcomes 163. Previous studies have demonstrated the utility of integrating AI-driven models with multi-omics and clinical data to improve prediction of atherosclerotic cardiovascular disease (ASCVD) risk and inform precision diagnostic and therapeutic strategies 164.

## Figures and Tables

**Table 1:** Key macrophage subsets, their markers and function in selected published studies on MI.

Publication	Disease	Species/Model	Macrophage subsets	Markers	Function
Dick, S. A. et al.^33^ (2019)	MI	Cx3cr1^CreER-YFP^R26^Td^ mice / LAD artery ligations model; Patients with end-stage cardiomyopathy during the time of implantation of the left ventricular assist device	Timd4 cluster	Timd4, Lyve1, Flor2	Tissue repair, inflammation resolution via IGF-1 secretion and efferocytosis.
			MHC-II^hi^ cluster	Cd14, Cx3cr1, Adgre1	Antigen presentation, tissue immune homeostasis.
			Ccr2+ cluster	Ccr2, Fcgr1, Plac8	Pro-inflammatory responses, contributing to ischemic damage.
			Isg cluster	Irf7, Isg20, Ifit1	Type I interferon signaling pathways, antiviral responses, inflammation regulation.
			proliferating	Mki67, Top2a	Proliferating.
			7 unique post-infarct macrophage clusters	Fn1, Il1b, Mmp14, Hif1a, Sirpb1a, Cd72, Lrg1, Trem2	Exhibiting plasticity but failing to fully adopt reparative functions. Contribute to heterogeneous pro-inflammatory and partial repair roles.
Farbehi, N. et al.^56^ (2019)	MI	Pdgfra^GFP/+^ mice / LAD artery ligations model	M1Mo	Ifitm6, Mcemp1	Phagocytose debris, secrete IL-1β/IL-6/TNFα, amplify acute inflammation.
			M1 Mφ	Arg1, C1qb, Ccr2, Ly6c2	Sustain inflammation via cytokine/chemokine secretion, promote leukocyte recruitment.
			M2 Mφ	Ly6c2-, Adgre1 high	Late-stage anti-inflammatory/reparative macrophages, secrete IL-10/TGF-β, resolve inflammation, promote angiogenesis, ECM remodeling and fibrosis resolution.
			MAC-TR	Timd4, Lyve1	Antigen presentation, phagocytosis, pro-regenerative roles, maintain cardiac homeostasis and fetal coronary development.
			MAC-IFNIC	Ifit3, Ifit1, Ifi47	IFN-γ/α/β-responsive subset, amplify inflammatory signaling, impair tissue repair.
			MAC6	Csf3r, Siglecf, S100a9	Granulocyte-enriched population, role in early inflammation unclear.
Bajpai, G. et al.^31^ (2019)	MI	Cx3cr1^CreER-YFP^: R26^Td^ mice / IR injury model	proliferating	Ki67, Ccnb2, Aurkb	Actively proliferate *in situ*, contribute to macrophage pool expansion post-injury.
			Tnip3+ cluster	Tnip3, ltgb7, Ltb4r1, ltgax	Unique cluster with dendritic cell-like features, role in immune regulation unclear.
			Lyve1+ cluster	Lyve1, CD163, Tim4, Lilra5	Regulate tissue homeostasis and repair.
			Fos+ cluster	Fos, Egr1, Hspa1a	Participate in inflammatory or stress responses.
			Mgst1+ cluster	Mgst1, Gpx3, Kif3a, Anpep	Anti-inflammatory, cell protection or homeostasis maintenance.
			Arg1+ cluster	Arg1, Adam8, Spp1	Anti-inflammatory/reparative phenotype, enriched in pathways for tissue repair and fibrosis.
			Cxcl1+ cluster	Cxcl1, Nlrp3, Ccrl2	Linked to adverse ventricular remodeling.
			Ifit3+ cluster	Ifit1-3, Mx1, lsg15	Associated with type I interferon signaling
Jung, S.-H. et al.^79^(2019)	MI	C57BL/6 mice / LAD artery ligations	steady-state (SS) Mφ1	Lyve1, F13a1, Cbr2, Cd163, Folr2	Tissue-resident macrophages, maintain homeostasis and prevent fibrosis.
			SS-Mφ2	H2-Eb1, H2-Aa, H2-Ab1, Cd74	Antigen-presenting resident macrophages.
			MI Early-Mφ	Cd68, Fcgr1, Itgam, Ccr2	Monocyte-derived pro-inflammatory macrophages, clear debris via phagocytosis.
			Late-Mφ 1	Apoe, Fcrls, Rgs10, Adgre1	Transitional macrophages with mixed inflammatory/repair functions.
			Late-Mφ 2	Trem2, Gpnmb, Fabp5, Spp1	Anti-inflammatory repair macrophages, promote tissue remodeling and angiogenesis.
			transient Mφ 1	Saa3, Fn1, Ltc4s	Phagocytic activity during mid-phase inflammation.
			transient Mφ 2	Fabp5, Spp1, Gpnmb	Early fibrotic signaling.
			transient Mφ 3	Hmox1, Prdx1, Gclm	Oxidative stress response.
			IFN- Mφ	Irf7, Isg15, Ifit2	Anti-viral response, modulate adaptive immunity.
			Proliferating Mφ	Top2a, Mki67, Hist1h1b	Self-renewal via local proliferation.
Rizzo, G. et al.^81^ (2022)	MI	C57BL/6 mice / LAD artery ligations; Patients with ischemic cardiomyopathy	RTM-TIMD4 cluster	Lyve1, Timd4, Folr2	Tissue-resident macrophages, self-renewing population, cardioprotective functions.
			RTM-MHCII cluster	MHCII, Mgl2	Tissue-resident macrophages, partially express CCR2, homeostatic maintenance.
			MHCII + Il1b + cluster	H2-Aa, Il1b, Tnip3, Tlr2, Tnfsf9, Axl	Pro-inflammatory phenotype, associated with tissue damage, NLRP3 inflammasome activity.
			Isg15^hi^ Mφ	Isg15、Irf7、Cxcl10, Il18	Type I interferon response, pathogenic inflammation, linked to IFNγ signaling.
			Trem2^hi^ Spp1^hi^ Mφ	Trem2, Spp1, Hmox1, Arg1	Monocyte-to-macrophage intermediate, profibrotic, efferocytosis activity.
			Trem2^hi^ Gdf15^hi^ Mφ	Trem2, Gdf15, Igf1, MerTk, Timp2, Apoe	Anti-inflammatory, tissue repair, cholesterol efflux regulation.
			Trem2^hi^ Prdx1^hi^ Mφ	Trem2, Prdx1	Iron-handling subset, antioxidant functions.
Amrute, J. M. et al.^82^ (2024)	MI	Six healthy donors, four patients with acute MI, six patients with ICM and six patients with NICM	CCR2+ Mφ	CCR2, IL-1β, CD68	Primary source of IL-1β signaling, drive FAP/POSTN fibroblast activation via IL-1β, co-localize with fibrotic niches in infarct zones.
			Inflammatory Mφ	SPP1, MMP9, CCL2	Mediate early inflammatory responses, promote extracellular matrix degradation, transition to profibrotic states.
			CCR2- Resident Mφ	FOLR2, LYVE1, MERTK	Associated with F4 fibroblasts (APOE/AGT), maintain tissue homeostasis, less involved in fibrotic remodeling.
Kuppe, C. et al.^84^ (2022)	MI	Four non-transplanted donor hearts (controls); Samples of patients with acute MI	LYVE+ PLTP+ Mφ	MAMDC2, PDE4D, SCN9A	Tissue-resident macrophages, associated with vascular homeostasis and tissue surveillance.
			LYVE+ FLOR+ Mφ	CD14, CX3CR1, ADGRE1	Tissue-resident macrophages, involved in immune regulation and tissue repair.
			SPP1+ Mφ	ITGAX, MMP19, SPP1	Phagocytotic activity, lipid uptake, and pro-fibrotic signaling, dominant in ischemic zones.
			CCL18+ Mφ	KCNMA1, HS3ST2, NHSL1, CPM	Linked to fibrotic remodeling and extracellular matrix deposition.
Xu, Y. et al.^77^ (2023)	MI	Cx3cr1^CreER-YFP^: R26^Td^ mice / LAD artery ligations model	Bhlhe41+ Mφ	Bhlhe41, Fabp5, Gpnmb, Cd36, Grn	Suppress myofibroblast activation, reduce fibrosis, limit infarct expansion.
			LYVE1+ MHCII- CCR2-Mφ	LYVE1, CCR2-	Homeostatic surveillance, decrease post-MI.
			LYVE1- MHCII+ CCR2+ Mφ	MHCII, CCR2+	Minor population at baseline, expand during inflammation.
			Trem2+ Spp1+ Mφ	Trem2+, Spp1+, IL-10+	Associated with late-stage remodeling, may promote fibrotic processes.
			Cluster 1	Tgfbi, Cd74, Ms4a4c, Ly6a	Pro-inflammatory response.
			Cluster 2	Il10, Spp1, Arg1, Cxcl3	Anti-inflammatory/repair functions.
			Mki67+ Mφ	Stmn1, Top2a, Mki67, Birc5	Active proliferation during early inflammation.
Ninh, V. K. et al.^76^ (2024)	MI	Ccr2^-/-^, Irf3^-/-^ mice; Patients with acute MI	IFNIC Mφ	ISGs (Ifit1, Rsad2, Cxcl10)	Localized to border zone IFNIC colonies; exhibit blunted pro-reparative functions due to type I IFN signaling, impairing fibroblast activation and matrix deposition.
			Resident Mφ	Timd4, Lyve1	Maintain tissue homeostasis, phagocytose debris, and initiate early repair responses in remote zones.
			Infiltrating Pro-inflammatory Mφ	Ccr2, Ly6c2, Chil3	Secrete pro-inflammatory cytokines, amplify sterile inflammation in the infarct zone.

**Table 2:** Key macrophage subsets, their markers and function in selected published studies on atherosclerosis.

Publication	Disease	Species/Model	Macrophage subsets	Markers	Function
Cochain, C. et al.^97^ 102 (2018)	Atherosclerosis	Ldlr^-/-^ mice / fed high-fat diet	Resident- like Mφ	Csf1r, Lyve1, Folr2, Pf4, Txnip	Exhibit anti-inflammatory/M2-like traits, may contribute to atherosclerosis via Pf4 and Txnip, potential roles in tissue homeostasis and lipid handling.
			Inflammatory Mφ	Cxcl2, Ccl3, Ccl4, Tlr2, Nlrp3	Proinflammatory phenotype and inflammasome activation, secrete chemokines to recruit immune cells, co-express feedback inhibitors to limit excessive inflammation.
			TREM2^hi^ Mφ	Term2, Cd9, Spp1	Lipid metabolism/catabolism specialization, enriched in oxidative stress response pathways.
Williams, J. W. et al.^103^ (2020)	Atherosclerosis	CX3CR1^creER/+^ Rosa26- lsl- ^Tomato^ Ldlr-/"	Adventitia Mφ	Lyve1, Mrc1 (CD206), Ccl2, Ccl6	Maintain arterial tone via ECM regulation, exhibit interferon signatures.
			Mac^AIR^	Il1b, Rgs1, Cd9, Mmp12, Acp5	First foam cells in early atherosclerosis, express IL-1β (primed inflammatory state), require CSF-1 for maintenance.
			Proliferating Mφ	Mik67, Ccna2, Top2a	Contribute to local expansion but insufficient for long-term plaque maintenance.
			Foamy Mφ	Trem2, SPP1, Fabp5, Gpnmb, Ctsd, Plin2	Converge from both Mac^AIR^ and monocyte origins, metabolic/storage phenotype, associated with plaque progression.
Zernecke, A. et al.^105^ (2023)	Atherosclerosis	12 scRNA-seq datasets of atherosclerotic mouse aortas; human carotid endarterectomy specimens from two studies	TLF-Cd209^hi^ Mφ	Lyve1, Timd4, Folr2, Cd209a/f/g	Tissue-resident macrophages, vascular homeostasis, lipid handling
			TLF-Cd209^low^ Mφ	Lyve1, Timd4, Folr2, Pf4, Mrc1	Adventitial-resident macrophages, tissue repair and immune surveillance
			Inflammatory Mφ	Nlrp3, Il1b, Ccl4, Ccl3	Pro-inflammatory response, NLRP3 inflammasome activation.
			CCR2^int^MHCII+	Ccr2, MHC-II genes (Cd74, H2-Eb1)	Transitional state between monocytes and macrophages, antigen presentation.
			Trem2^hi^-Slamf9	Trem2, Slamf9, Ch25h, Tnf	Early lipid-loaded macrophages, inflammatory lipid metabolism.
			Trem2^hi^-Gpnmb	Trem2, Gpnmb, Spp1, Apoe, Fabp5	Foamy macrophages, lipid accumulation, cholesterol efflux, lesion progression.
			Mac^AIR^ Mφ	Acp5, Gngt2, MHC-II	Intimal-resident macrophages, self-renewal, vascular barrier maintenance.
			IFNIC Mφ	Isg15, Oasl2, Irf7	Type I interferon response, antiviral defense.
			hInflammator Mφ	CD74, HLA-DRB1, IL1B, CXCL8	Pro-inflammatory cytokine secretion, plaque destabilization.
			hFoamy Mφ	TREM2, SPP1, APOE, FABP5, GPNMB	Lipid metabolism, plaque core formation.
			hIFNIC Mφ	ISG15、IFI6、MX1	Type I interferon response, immune activation.
			hProlif	TUBB、H2AFZ、STMN1	Proliferating.
			hLYVE1-Mφ	LYVE1, MRC1, FOLR2, SEPP1	Tissue-resident macrophages, vascular repair, matrix remodeling.
van Kuijk, K. et al.^106^ (2022)	Atherosclerosis	PHD^ko^ mic / fed high cholesterol diet; Human plaque tissue	Resident-like Mφ	Timd4, Lyve1, Flor2	Maintain tissue homeostasis, may represent steady-state macrophages in early lesions.
			Inflammatory Mφ	Tnip3, Nlrp3, Tnfsf9, Cxcl10	Pro-inflammatory, associated with plaque inflammation.
			Trem2^hi^ Mφ	Term2, Mmp12, Spp1	Foamy phenotype with enhanced fibrotic signaling, secrete Spp1 to activate fibroblasts for collagen production.
			IFNIC Mφ	Isg15, Ifit3, Irf7	Respond to interferon signaling, potential role in antiviral-like responses.
			Cavity Mφ	Fn1, Gata6, Vsig4+	Resemble peritoneal cavity macrophages, proposed role in debris clearance and lipid handling.
Bashore, A. C. et al.^107^ (2024)	Atherosclerosis	Patients with carotid atherosclerosis	IL1B+ Mφ	IL1B, NLRP3, CCR2 (CD192), CD64, CD11c	Pro-inflammatory, NLRP3 inflammasome activation, key drivers of plaque inflammation, potential targets for IL-1β inhibitors.
			C1Q+ Mφ	C1Q genes (C1QA, C1QB, C1QC), CD64, CD11c, MHCII	Complement activation, anti-inflammatory, efferocytotic capacity, enriched in STAT1/RELA-mediated immune regulation.
			Apoptotic Mφ	Granzyme A, mitochondrial genes	Apoptotic cell clearance, high oxidative stress markers, phagocytic clearance functions.
			Foam Mφ1 (TREM2+)	TREM2, ABCA1, LPL, CD36, APOE	Lipid metabolism specialists, cholesterol efflux, potential plaque-stabilizing functions, may represent homeostatic foam cells.
			Foam Mφ2 (Inflammatory)	TREM2, APOE, C1Q genes, CCL18	Hybrid phenotype (foamy + inflammatory), lipid processing with residual inflammation.
			Proliferative Mφ	MKI67, TUBB, STMN1	MYC-driven cell cycling, high DNA damage signaling, may contribute to macrophage persistence.
			ACTA2+ Mφ	MYH11, ACTA2, MYOCD, CNN1	Express smooth muscle cell genes, fibrotic/EMT pathway activation, possibly SMC-derived transdifferentiated cells, SMAD3/MRTF-mediated regulation.
Cai, J. et al.^108^ (2020)	Atherosclerosis	allograft-induced transplant arteriosclerosis mouse	Resident- like Mφ	Mrc1, Folr2	Anti-inflammatory, phagocytosis of apoptotic cells, tissue homeostasis.
			Inflammatory Mφ	Eno1, Tpi1, Prdx5, Ccl2, Ccl7	Proinflammatory response, secretion of chemokines, glycolysis and ROS production.
			ISG^hi^ Mφ	H2-Ab1, H2-Aa, Ifitm3, Ifitm6	Strong interferon response with antigen presenting capacity.
			Lgals3^hi^ Mφ	Lgals3	Expressed Plin2, a critical regulator in foam cell formation in atherosclerosis.
Depuydt, M. A. C. et al.^111^ (2020)	Atherosclerosis	Patients with atherosclerosis	Cluster 0 (IL1B+ Mφ)	IL1B, CASP1, CASP4	Pro-inflammatory phenotype with inflammasome activation, leukocyte transendothelial migration.
			Cluster 1 (TNF+ Mφ)	TNF, TLR4, CCL3, CXCL1	Pro-inflammatory macrophages driven by TNF and IFN signaling, expressed Toll-like receptors, IFNγ-driven activation via T-cell interactions.
			Cluster 2 (Foam Mφ)	TREM2, ABCA1, ABCG1, MMP9	Lipid metabolism and fibrosis promotion, anti-inflammatory via LXR/RXR pathway activation, expressed smooth muscle markers (partial), suggesting fibrotic plasticity.
			Cluster 3 (Dendritic-like Mφ)	CD1C, CLEC10A, HLA-DR	Enhanced antigen presentation and IL12 production, drives T-cell activation.
			Cluster 4	CD3E, FOXP3 (misclassified)	Likely a misclustered population containing regulatory T cells.
Fernandez, D. M.^112^ (2019)	Atherosclerosis	Patients with atherosclerosis	Cluster 1	HLA-DRA, CD74	Activated macrophages, antigen presentation and inflammatory responses.
			Cluster 2 (Pro-inflammatory)	CYBA, LYZ, S100A9, S100A8, TIMP1	Highly inflammatory, promotes oxidative stress and TLR signaling, reduces ECM degradation via TIMP1.
			Cluster 3	JUNB, NFKBIA, MALAT1	Pro-inflammatory, activates NF-κB signaling, associated with cholesterol efflux (LXR/RXR pathways).
			Cluster 5 (Foam cells)	APOC1, APOE, PLIN2	Lipid-laden foam cells, cholesterol uptake, metabolism, and lipid accumulation, anti-inflammatory.

**Table 3:** Key macrophage subsets, their markers and function in selected published studies on HF.

Publication	Disease	Species/Model	Macrophage subsets	Markers	Function
Martini, E. et al. 119 (2019)	HF	C57BL/6J mice / TAC model	Timd4^hi^MHCII^hi^ Mφ	Mgl2, Timd4, MHCII	Promote angiogenesis and reduce fibrosis, maintain cardiac homeostasis.
			Timd4^hi^ (Lyve1^hi^) Mφ	Timd4, Lyve1, Ccr2-	Phagocytosis of apoptotic cells, proliferate in response to stress, protect against adverse remodeling.
			MHCII^hi^ Mφ	MHCII, Ccr2+	Pro-inflammatory roles, increase during early stress but decline in late heart failure.
			Double-Negative Mφ	Low/negative for Timd4, MHCII	Function unclear, may represent transitional or precursor states.
Revelo, X. S. et al^120^ (2021)	HF	C57BL/6J mice, CCR2^KO^ mice / TAC model	CCR2- Resident Mφ	Timd4, Lyve1, Cd163	Promote angiogenesis, inhibit fibrosis, maintain tissue homeostasis, regulate cardiac conduction and metabolic stability.
			CCR2+ monocyte-derived Mφ (MoMFs)	MHC-II, Ccr2, Ccl5, Cxcl10, Il1b, Spp1, Thbs1, Fn1	Promote proinflammatory responses, drive fibrosis, recruit monocytes and T cells.
			Cluster 0 (MoMFs)	Ifit1, Cxcl10	Recruit monocytes and Th1 cells via Cxcl10.
			Cluster 3 (Resident)	Myd88 pathway genes	Mediate innate immune signaling.
			Cluster 4 (Resident)	Ptder4	Regulate prostaglandin-mediated tissue repair.
			Cluster 5 (MoMFs)	Spp1	Promote fibrotic remodeling through osteopontin.
			Cluster10 (MoMFs)	Ccl5, Cxcl16	Enhance inflammation and leukocyte recruitment.
			Cluster14 (MoMFs)	Thbs1, Fn1	Drive ECM deposition and fibrosis.
Ni, S.-H. et al.^121^ (2022)	HF	C57BL/6 mice; TAC and chronic Ang II infusion; patients with DCM	CD72hi Mφ	Cd72, Ccr2	Pro-inflammatory phenotype, induce cardiomyocyte apoptosis, promote oxidative stress via ROS production, aggravate cardiac injury.
Rao, M. et al.^122^ (2021)	HF	Patients with DCM and ICM who were undergoing heart transplantation	CCR2-HLA-DR^hi^ C1	LYVE1, LILRB5, MAF, SIGLEC1, SLC40A1, BLVRB, STAB1, DAB2	Tissue-resident macrophages enriched in mild-lesion/normal hearts, negative immunomodulation, maintain tissue homeostasis.
			CCR2-HLA-DR^hi^ C2	CLEC4E, CLEC7A, CLEC10A	Tissue-resident macrophages, enriched in pattern recognition receptor signaling, may mediate pathogen sensing and immune surveillance.
			CCR2+HLA-DR^hi^ C1	C1QA, C1QB	Tissue-resident macrophages enriched in severely fibrotic regions, exhibit phagocytic activity, antigen clearance and immune regulation.
			CCR2+HLA-DR^hi^ C2	CXCL8, NLRP3, BIRC3, EGR1	Pro-inflammatory subset enriched in fibrotic regions, activate NLRP3 inflammasome and NF-κB signaling, recruit leukocytes and promote inflammation, interact with activated endothelial cells via CXCL8-DARC axis.
			CCR2+HLA-DR^hi^ C3	CCR2, S100A8, S100A9	Infiltration-derived macrophages, transition into CCR2⁺HLA-DR⁺ⁱ C2 via ATF3/KLF4 regulons, contribute to pro-inflammatory responses.
			TREM2+ Mφ	TREM2, SPP1, LGALS3	Angiogenesis and immune suppression, intermediate state with reduced inflammatory response compared to CCR2⁺HLA-DR⁺ⁱ subsets.
Amrute, J. M. et al.^123^ (2023)	HF	Patients with HF who experienced either cardiac recovery or ongoing HF before and after LVAD implantation	Mφ1	SPP1, FN1, TPRG1, PPARG	Matrix remodeling, may contribute to pathological tissue changes.
			Mφ2	NAMPT, NR4A1, PLAUR, FOSB	Pro-inflammatory, negative prognosis for cardiac recovery, monocyte-derived inflammatory macrophages.
			Mφ3	CXCL10, CCL4, ENOX1, GBP1, BIRC3	Promote inflammation and respond to interferon signaling.
			Mφ4	IFI44L, MX1, EPSTI1	Interferon-responsive state, unresolved inflammation.
			Mφ5	NAV2, SCN9, ARNF150, MAMDC2	Resident macrophages, tissue repair and remodeling.
			Proliferating Mφ	DIAPH3, ARHGAP11B, ATAD2	Proliferating.

**Table 4:** Key macrophage subsets, their markers and function in selected published studies on myocarditis and CS.

Publication	Disease	Species/Model	Macrophage subsets	Markers	Function
Hua, X. et al.^128^ (2020)	Myocarditis	Balb/c mice / Autoimmune myocarditis (EAM model)	Cluster 2	Mlr1, Cxcl9, Ly6i, Nos2, Arg1	Oxidative phosphorylation, antigen processing/presentation, nitric oxide biosynthesis, dominant in acute inflammation.
			Cluster 3	Ccl8, Fbx32, Hif1a, Rxra	Antigen presentation, neutrophil degranulation, peak in subacute phase.
			Cluster 7	Ccr7, Clec10a, Tnip3	IL-10-mediated anti-inflammatory responses, wound healing, dominant in myopathy phase.
			Cluster 8	Tnf, Il-10, Vsig4	Immune regulation through IL-10 signaling, increased in chronic phase.
Hu, Z. et al.^129^ (2023)	Myocarditis	Lewis rats / Giant cell myocarditis model	Cluster 1	Prdx1, Prdx5, Arg1, Ass1	High IFN-γ stimulated gene scores, enhanced phagocytosis and inflammation, autoimmunity.
			Cluster 2	Ms4a7, Tmem176a, Tmem176b	Expressed MS4A family genes, involved in antigen processing/presentation.
			Cluster 3	Nr4a1	Tissue-resident macrophages, regulates immune response.
			Cluster 4	Pf4	Pleiotropic chemokine expression, limits macrophage activation, recovery processes.
			Cluster 5	Fcnb, Plac8, Vcan	Tumor necrosis factor signaling, interferon/cytokine signaling, pro-inflammatory functions.
Pan, M. et al. 131 (2024)	Myocarditis	Ctla4+/+Pdcd1-/-, Ctla4+/-Pdcd1-/- mice	Cd163+ resident Mφ	Cd163, Lyve1, Folr2, Cbr2	Tissue-resident macrophages, homeostatic maintenance, potential roles in stress response and tissue repair.
			Cxcl9+Cxcl10+ Mφ	Cxcl9, Cxcl10, Gbp2b, Fcgr4, CCR2	IFN-γ-activated inflammatory macrophages, secretion of chemokines, antigen presentation, T-cell recruitment/activation via CXCR3 signaling, antibody-dependent cytotoxicity (ADCC) potential.
			Nlrp3+ Mφ	Nlrp3, Ccl4, Cd14	Pro-inflammatory, inflammasome activation, IL-1β production, stromal interaction.
Liu, J. et al.^132^ (2022)	CS	patients with cardiac sarcoidosis	GPNMB+ Mφ	GPNMB, TPRG1, SNTB1	Lysosomal biogenesis, PPAR pathway activation, giant cell differentiation via MITF/TFEC regulation, granuloma structural organization.
			HLA-DR+ Mφ	HLA-DR, CIITA, IL12RB1	Antigen presentation, mTOR pathway activation, interface between giant cells and immune microenvironment.
			SYTL3+ Mφ	SYTL3, FCGR3A, IL18R1	Pro-inflammatory, mTOR-driven proliferation, intermediate differentiation state, phagosome/ECM remodeling.
			Resident Mφ	CD163, F13A1, SCN9A	Tissue homeostasis, low inflammatory activity.

**Table 5:** Major macrophage subtypes, their biomarkers, and functions in MI, Atherosclerosis and HF.

Major Macrophage Subset	Key Markers	Functions
Timd4+/Lyve1+ Mφ	Timd4, Lyve1, Folr2	In MI: Maintain cardiac homeostasis, promote repair, IGF-1 secretion, modulate inflammation
		In atherosclerosis: Maintain vascular homeostasis, anti-inflammatory, lipid handling to reduce arterial stiffness
		In HF: Phagocytosis and clearance of apoptotic cells, antifibrotic, maintain heart function
MHCII^hi^ Mφ	MHC-II, Cd74, H2-Ab1, H2-Eb1, Cx3cr1, Adgre1	In MI: Antigen presentation, immune regulation, involved in repair
		In atherosclerosis: Antigen presentation, partly pro-inflammatory
		In HF: Antigen presentation, tissue repair, immune surveillance
CCR2+ Monocyte-derived Mφ	CCR2, Ly6C, FCGR1, Ccl2, Plac8, Osm	In MI: Pro-inflammatory responses, clear necrotic debris, promote fibrosis, recruit immune cells
		In atherosclerosis: Pro-inflammatory, promote plaque progression, lipid deposition
		In HF: Pro-inflammatory, drive cardiac fibrosis and dysfunction
Trem2^hi^ Mφ	Trem2, Gpnmb, Fabp5, Apoe, Arg1, Mmp12	In MI: Anti-inflammatory/reparative, promote remodeling, clear apoptotic and dysfunctional mitochondria
		In atherosclerosis: Foam cells with lipid metabolism, promote cholesterol efflux and fibrosis progression
		In HF: Promote angiogenesis and immune suppression, reduce inflammation
ISG+ / IFNIC Mφ	Isg15, Ifit1/2/3, Irf7, Rsad2, Cxcl10	In MI: Regulate antiviral and inflammatory responses, partially impair repair
		In atherosclerosis: Promote inflammation and antiviral responses in plaque
		In HF: May promote persistent inflammation, negative prognosis
Proliferating Mφ	Mki67, Top2a, Ccnb2, Birc5, Tubb	In MI: Expand early inflammatory macrophage pool
		In atherosclerosis: Support local expansion of foam cells
		In HF: Expand macrophage populations early in remodeling
CD72^hi^ Mφ	Cd72, Ccr2	In MI: -
		In atherosclerosis: -
		In HF: Pro-inflammatory, induce cardiomyocyte apoptosis and oxidative stress, worsen cardiac injury
Mac^AIR^ Mφ	Il1b, Rgs1, Cd9, Mmp12, Acp5, MHC-II	In MI: -
		In atherosclerosis: Foam cell precursors in early plaque, maintain vascular barrier
		In HF: -

## Abbreviations

CVD: cardiovascular diseases
ECM: extracellular matrix
scRNA-seq: single-cell RNA sequencing
ST-seq: spatial transcriptomics sequencing
MI: myocardial infarction
TLRs: toll-like receptors
LPS: lipopolysaccharide
IFN-γ: interferon gamma
iNOS: inducible nitric oxide synthase
IL-4: interleukin-4
IL-13: interleukin-13
ARG1: arginase 1
YM1: chitinase-like protein 3
PGC-1β: PPARγ coactivator-1β
TCA: tricarboxylic acid cycle
HIF1α: hypoxia-inducible factor 1 alpha
TREM2: triggering receptor expressed on myeloid cells 2
Bhlhe41: basic helix-loop-helix family member e41
NLRP3: NOD-like receptor protein 3
MHCII: major histocompatibility complex class II
LYVE1: lymphatic vessel endothelial hyaluronan receptor 1
FOLR2: folate receptor 2
CCR2: C-C chemokine receptor type 2
HLA-DRA: human leukocyte antigen-DR alpha
HLA-DMA: human leukocyte antigen-DM alpha
HLA-DMB: human leukocyte antigen-DM beta
HLA-DPA1: human leukocyte antigen-DP alpha 1
IL4R: interleukin-4 receptor
STAT3: signal transducer and activator of transcription 3
ITGAM: integrin alpha M
C1QA: complement C1q A chain
SAN: sinoatrial node
AVN: atrioventricular node
IGF1: insulin-like growth factor 1
BMDMs: bone marrow-derived macrophages
TGF-β: transforming growth factor-beta
Smad3: SMAD family member 3
IL-1β: interleukin-1 beta
NF-κB: nuclear factor kappa B
SPP1: secreted phosphoprotein 1
CD44: cluster of differentiation 44
SYK: spleen tyrosine kinase
TNF: tumor necrosis factor
CCL4: C-C motif chemokine ligand 4
HLA-DR: human leukocyte antigen-DR
PD-1: programmed cell death protein 1
PD-L1: programmed death-ligand 1
CTLA-4: cytotoxic T-lymphocyte associated protein 4
CXCL9: C-X-C motif chemokine ligand 9
CXCL10: C-X-C motif chemokine ligand 10
CXCR3: C-X-C chemokine receptor type 3
CCL2: C-C motif chemokine ligand 2
MCP1: monocyte chemoattractant protein 1
CCL7: C-C motif chemokine ligand 7
MCP3: monocyte chemoattractant protein 3
ADCC: antibody-dependent cellular cytotoxicity
STAT1: signal transducer and activator of transcription 1
JAK2: janus kinase 2
GLUT1: glucose transporter 1
GLUT4: glucose transporter 4
ROS: reactive oxygen species
EGFR: epidermal growth factor receptor
PDGFRA: platelet-derived growth factor receptor alpha
PDGFC: platelet-derived growth factor C
GPNMB: glycoprotein non-metastatic melanoma protein B
MITF: microphthalmia-associated transcription factor
CRISPR: clustered regularly interspaced short palindromic repeats
Cas9: CRISPR-associated protein 9
AI: artificial intelligence, ML: machine learning
MALDI-MSI: matrix-assisted laser desorption/ionization mass spectrometry imaging
IMC: imaging mass cytometry
MSI: mass spectrometry imaging
MERFISH: multiplexed error-robust fluorescence *in situ* hybridization
